# One-Step Synthesis of Au-Ag Nanowires through Microorganism-Mediated, CTAB-Directed Approach

**DOI:** 10.3390/nano8060376

**Published:** 2018-05-28

**Authors:** Luhang Xu, Dengpo Huang, Huimei Chen, Xiaoling Jing, Jiale Huang, Tareque Odoom-Wubah, Qingbiao Li

**Affiliations:** 1 Department of Chemical and Biochemical Engineering, College of Chemistry and Chemical Engineering, and National Laboratory for Green Chemical Productions of Alcohols, Ethers and Esters, Xiamen University, Xiamen 361005, China; 20620151152241@stu.xmu.edu.cn (L.X.); 20620121151383@stu.xmu.edu.cn (D.H.); 33120120153552@stu.xmu.edu.cn (H.C.); cola@xmu.edu.cn (J.H.); kelqb@xmu.edu.cn (Q.L.); 2 Department of Chemistry, College of Chemistry and Chemical Engineering, and National Laboratory for Green Chemical Productions of Alcohols, Ethers and Esters, Xiamen University, Xiamen 361005, China; 3 College of Chemical Engineering and Material Science, Quanzhou Normal University, Quanzhou 362002, China

**Keywords:** microorganism-mediated, Au-Ag NWs, SERS enhancement, *Pichia pastoris* cells, CTAB

## Abstract

Synthesis and applications of one dimensional (1D) metal nanostructures have attracted much attention. However, one-step synthesis of bimetallic nanowires (NWs) has remained challenging. In this work, we developed a microorganism-mediated, hexadecyltrimethylammonium bromide (CTAB)-directed (MCD) approach to synthesize closely packed and long Au-Ag NWs with the assistance of a continuous injection pump. Characterization results confirmed that the branched Au-Ag alloy NWs was polycrystalline. And the Au-Ag NWs exhibited a strong absorbance at around 1950 nm in the near-infrared (NIR) region, which can find potential application in NIR absorption. In addition, the Au-Ag NWs showed excellent surface-enhanced Raman scattering (SERS) enhancement when 4-mercaptobenzoic acid (MBA) and rhodamine 6G (R6G) were used as probe molecules.

## 1. Introduction

Synthesis of one dimensional (1D) nanostructures has been in the limelight in the past decade [1,2]. For example, fascinating developments have been achieved in the synthesis of metal nanowires (NWs) which represents an important class of 1D nanostructures. Metal NWs have a transverse surface Plasmon resonance (TSPR) absorption peak and a longitudinal surface Plasmon resonance (LSPR) absorption peak, corresponding to the longitudinal oscillation of electrons and the transverse electronic oscillation, respectively [3]. Compared to bulk metal, metal NWs have excellent dimensional effects, and they are able to form more complex nanoscale structures [4,5,6].

In contrast to their monometallic counterparts, bimetallic NWs are generally synthesized through galvanic replacement reactions in which the second metal precursor is reduced by monometallic NWs pre-synthesized from the first metal precursor [7]. Alternatively, they can be prepared by reducing two metal precursors simultaneously with the sacrificial NWs premade from the third metal precursor [8]. However, the two-step protocols may lead to severe metal leaching during the replacement reactions. Moreover, the pre-synthesized or sacrificial NWs are generally synthesized under harsh conditions [9]. To date, one-step synthesis of bimetallic NWs has remained a challenging issue.

The surface structure in non-enzymatic microorganisms provided an excellent bio-templating medium to grow metal nanostructures [10,11,12]. In our previous study, we described a one-step microorganism-mediated, surfactant-directed approach to bimetallic AuPd nanoflowers in the presence of cetyltrimethylammonium chloride (CTAC) at room temperature [7]. Furthermore, metal leaching can be effectively overcome by the one-step synthesis [7]. In addition, the AuPd nanoflowers can form functional nanocomposites with microorganisms [7]. Herein, instead of CTAC, a microorganism-mediated, hexadecyltrimethylammoniumbromide (CTAB)-directed (MCD) approach was expanded to one-step synthesis of Au-Ag NWs. Herein, chloroauric acid (HAuCl_4_) and silver nitrate (AgNO_3_) were selected as metal precursors, while ascorbic acid (AA) was selected as reductant. The previous *Pichia pastoris* cells (PPCs) were employed, which were dried and milled into easily preserved cell powder. Moreover, continuous injection of AgNO_3_ solution was adopted to suppress the formation of AgBr precipitate. It was very interesting that Au-Ag NWs were facilely tuned. Scanning electron microscopy (SEM), transmission electron microscopy (TEM), X-ray diffraction (XRD), flame atomic absorption spectrophotometer (AAS) and selected-area electron diffraction (SAED) were used to characterize Au-Ag nanostructures. Diffuse reflectance ultraviolet-visible (DRUV-Vis) and surface enhanced Raman scattering (SERS) were used to examine the optical properties of Au-Ag NWs. Interestingly, the as-obtained Au-Ag-NW/PPC nanocomposites can be directly used for SERS detection of some probe molecules.

## 2. Experimental Section

### 2.1. Experimental Materials

Ascorbic acid (AA, 99%) was purchased from Sangon Biotech (Shanghai Co., Ltd., Shanghai, China). Chloroauric acid (HAuCl_4_·3H_2_O, 99.99%), silver nitrate (AgNO_3_, 99.99%) and hexadecyltrimethylammonium bromide (CTAB, 99.9%) were purchased from Sinopharm Chemical Reagent Co., Ltd., Shanghai, China. And 4-mercaptobenzoic acid (MBA) and Rhodamine 6G Molecule were purchased from Aladdin Reagents (Shanghai). All chemicals were used as received without further purification.

### 2.2. Synthesis of Au-Ag NWs

**Figure 1:**
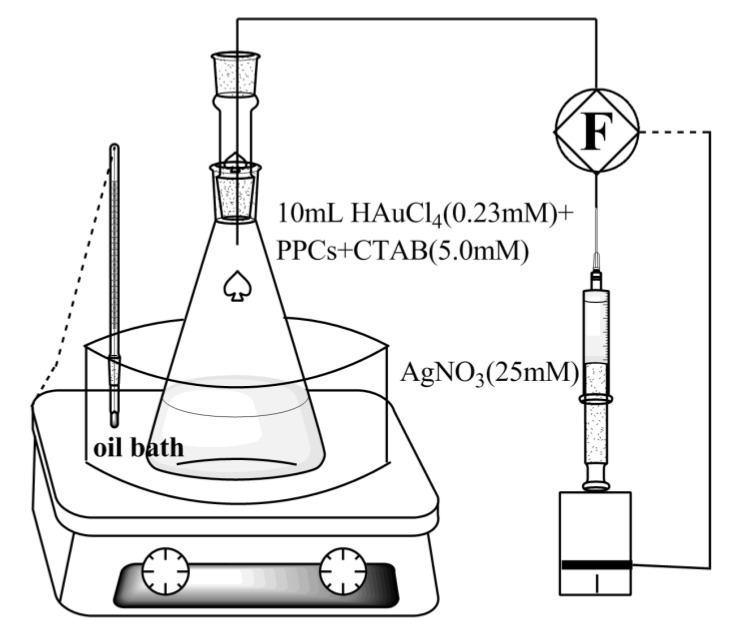
Reaction equipment.

The PPCs were cultivated and the PPC powder was prepared according to the procedures reported in our previous work [13]. The comprehensive procedure is written in the supporting information (SI). For the synthesis of Au-Ag NWs, PPCs were initially added to aqueous CTAB. Aqueous HAuCl_4_ was added to the initial reaction solutions in Erlenmeyer flask with a total volume of 10 mL. The solutions were then put in an oil bath (30–90 °C) under magnetic stirring. The cells interacted with Au (III) for 15 min before the AA solution was added. As shown in Figure 1, while AA was added, an injection pump was used to add aqueous silver nitrate (10 mL) into the initial reaction solution. Thereby, the formation of AgCl and AgBr precipitate can be avoided. The injection rate of aqueous AgNO_3_ is 1 mL·h^−1^. Some precipitates were observed at the bottom of the Erlenmeyer flask after 10 h. The resulting solutions were sampled and centrifuged at 2000 rpm for 10 min. Thereafter, the supernatant was decanted, and the precipitates were dispersed in 200 µL of deionized water.

### 2.3. Characterization of Au-Ag NWs

The TEM samples of Au-Ag NWs were obtained by dropping hydrosol on carbon-coated copper grids. TEM observation was performed using a Tecnai F30 Microscope (FEI, Amsterdam, The Netherlands). The SEM samples of the suspension were prepared by dropping the suspension (containing spontaneously formed precipitates) onto clean silicon and allowing water to evaporate completely. SEM observations were carried out using an LEO-1530 Electron Microscope (LEO, Oberkochen, Germany). After the reaction, the resulting solutions were centrifuged at 2000 rpm for 10 min, and the precipitates were dried at 30 °C. The dried mixtures were collected, and an X’Pert Pro X-ray Diffractometer (PANalytical BV, Almelo, The Netherlands) operated at 40 kV and 30 mÅ with Cu-Kα radiation was used to determine the formation of Au-Ag NWs. The dried mixtures were scanned from 300 to 2500 nm by UV-Vis-NIR spectrometer (Cary 5000, Agilent, CA, USA) to obtain DRUV-Vis absorption spectra of the Au-Ag NWs. Flame atomic absorption spectrophotometer was used to measure the absorbance value of Au and Ag in the solution calculating concentration of Au and Ag. For SERS enhancement of the Au-Ag NWs, 0.005 g of the Au-Ag-NW/PPC composites was dried on a glass slide after the Au-Ag NWs were washed with deionized water. Then 30 µL R6G or MBA was dropped on the composites for Raman measurement. The Raman spectra were recorded on a Renishaw in via spectrometer with a 632.8 nm He-Ne laser as the excitation source [13]. The laser power that reached the sample was 0.4 mW. The acquisition time was 10 s, and the spectra were obtained once. A minimum of three samples were tested for each concentration. However, no marked difference was observed between the data.

## 3. Results and Discussion

### 3.1. Characterization of Au-Ag NWs

**Figure 2:**
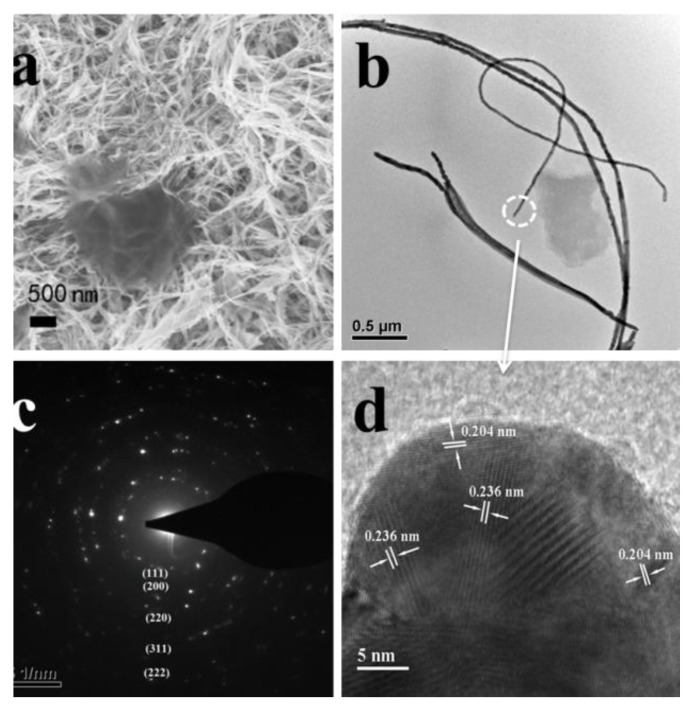
(**a**) Scanning electron microscopy (SEM) image; (**b**) transmission electron microscopy (TEM) image; (**c**) selected-area electron diffraction (SAED) pattern and (**d**) High-resolution TEM (HRTEM) image of Au-Ag/nanowires (NWs).

**Figure 3:**
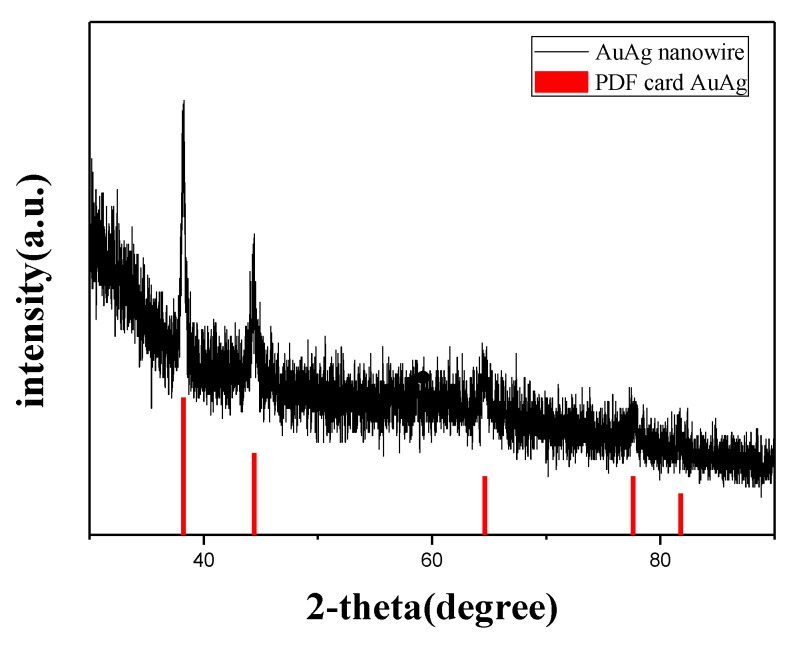
X-ray diffraction (XRD) pattern of Au-Ag NWs.

Well-defined and closely packed Au-Ag NWs (Figure 2) could be synthesized through the reduction of aqueous HAuCl_4_ (0.23 mM) and AgNO_3_ (0.23 mM, 1 mL·h^−1^) with AA (0.5 mM) in the presence of CTAB (9.0 mM) and PPCs (0.005 g) at 30 °C. Au-Ag-NW/PPC composites precipitated at the bottom of the solution. The TEM and SEM images (Figure 2a,b) showed the product was nanowires. The typical SAED pattern (Figure 2c) from single Au-Ag NW in Figure 2b showed Bragg reflections of {111}, {200}, {220}, {311} and {222}, indicating that the Au-Ag NWs was polycrystalline. The High-resolution TEM (HRTEM) image (Figure 2d) showed the lattice spacing of two crystal planes in Au-Ag NWs were 0.204 nm and 0.236 nm, which corresponds to that of the Au-Ag (111) and Au-Ag (200) crystal planes. A couple of obvious Bragg reflections (2θ = 38.2°, 44.4°, 64.6° and 77.6°) were noticeably exhibited in the XRD pattern of crystalline Au-Ag NWs (Figure 3), which may be indexed based on the face-centered-cubic structure of Au-Ag. The four Bragg reflections may be corresponding to {111}, {200}, {220}, {311} crystal planes of the face-centered-cubic structure of Au-Ag, respectively.

**Figure 4:**
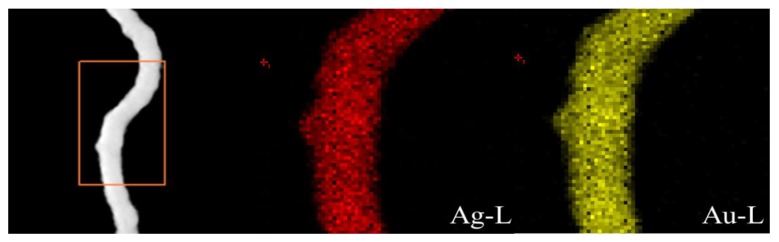
Scanning transmission electron microscope mapping (STEM) image of the single Au-Ag NW synthesized at 30 °C.

**Figure 5:**
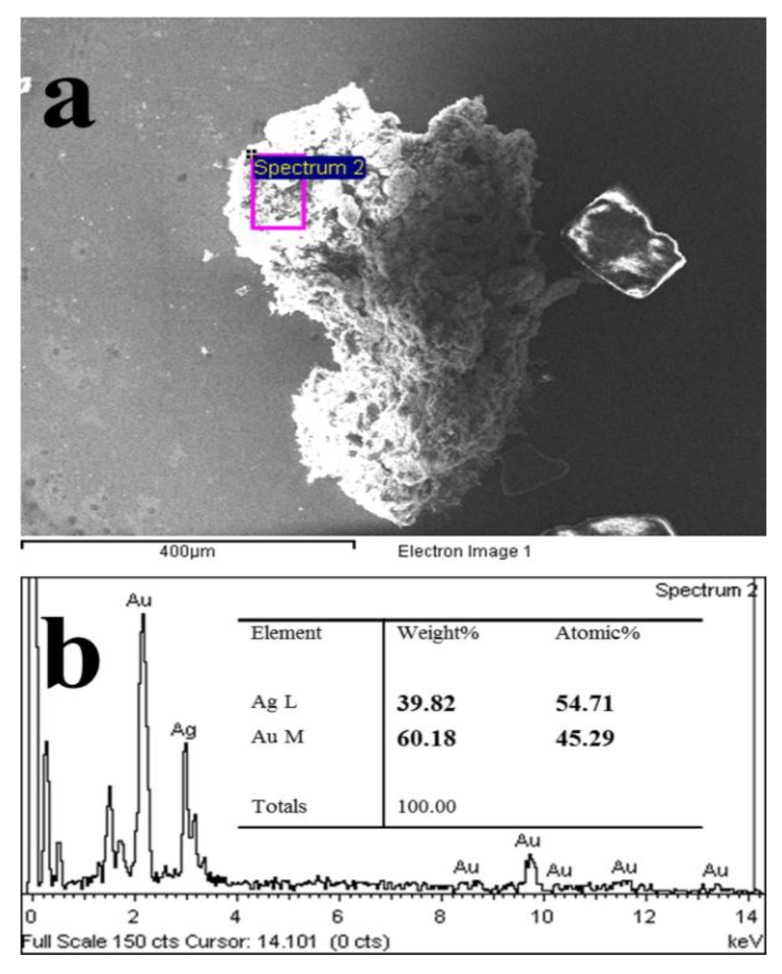
(**a**) Au-Ag NWs showing the square scanned area and (**b**) Energy-dispersive X-ray EDX spectra of the Au-Ag NWs.

To further verify the structure of Au-Ag NWs, the Scanning transmission electron microscope (STEM) mapping was used to analyze the elemental distribution of Au and Ag in the Au-Ag NWs. As showed in Figure 4, Au and Ag uniformly distributed in the entire nanowires and there was no clear boundary between Au and Ag. Obviously, the nanowires were structurally alloy. Furthermore, the Energy-dispersive X-ray (EDX) spectrum of Au-Ag NWs was carried out, as shown in Figure 5. The nanowire (square area) was scanned to analyze the contents of Au and Ag. It was found that the mole percent of Au and Ag were 45.29% and 54.71%. In other words, the ratio of Au-Ag is closed to 1:1, which is almost the same with the initial ratio of the two precursors.

However, when *Escherichia coli* cells (*E. coli*) is used instead of PPCs, as seen in Figure S1, thick chain-like Au-Ag structures are observed possibly formed through the interconnection of Au-Ag particles and short rod-like structures. Meanwhile, in the absence of PPCs Au-Ag nano-belts are observed. And as such the long Au-Ag NWs were impossible to produce without PPCs.

### 3.2. Effect of Temperature on Au-Ag NWs

**Figure 6:**
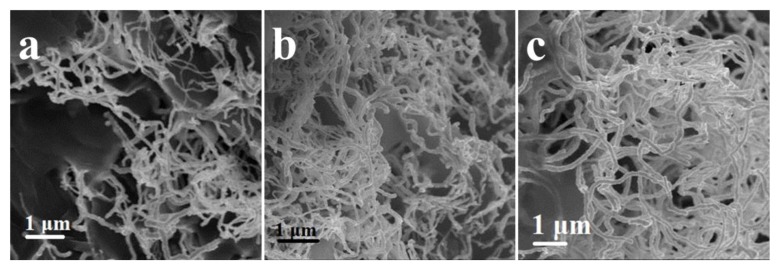
The SEM images of Au-Ag NWs synthesized at (**a**) 30 °C; (**b**) 60 °C; (**c**) 90 °C.

Well-defined and closely packed Au-Ag NWs could be synthesized through the reduction of aqueous HAuCl_4_ (0.23 mM) and AgNO_3_ (0.23 mM, 1 mL·h^−1^) with AA (0.5 mM) in the presence of CTAB (9.0 mM) and PPCs (0.005 g) at 30 °C, 60 °C and 90 °C, shown in Figure 6. The nanowires synthesizedat30 °C, 60 °C and 90 °C were all well-defined, and their morphologies were identical with similar diameter. It was found that the effect of Au-Ag NWs did not depend on reaction temperature. To reduce the energy consumption, the reaction temperature of 30 °C was chosen for the follow-up experiments.

### 3.3. Formation Mechanism of Au-Ag NWs

**Figure 7:**
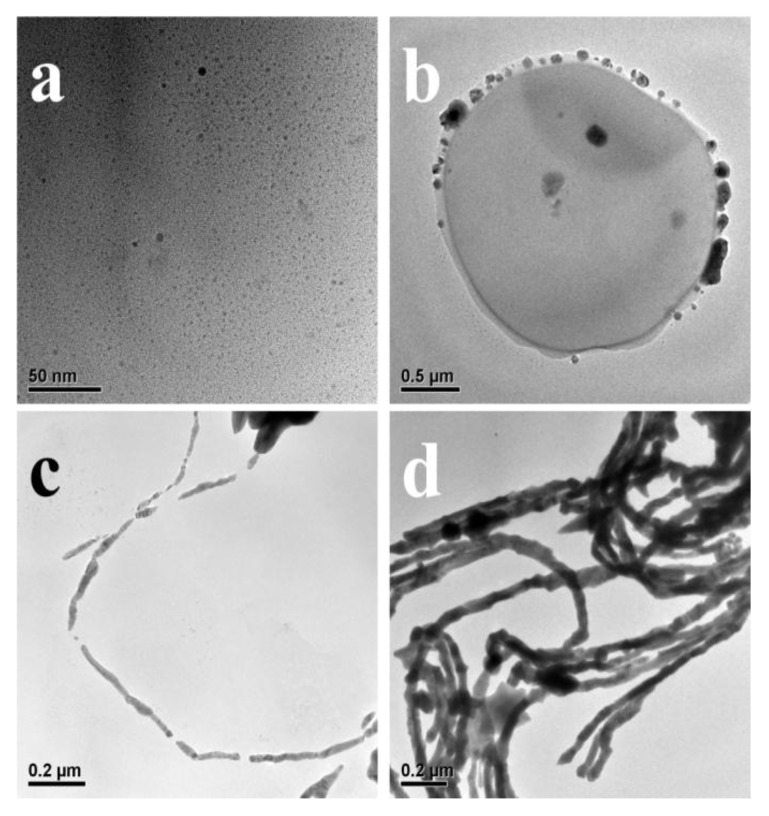
TEM images of Au-Ag nanostructures synthesized through the reduction for (**a**) 0 min; (**b**) 10 min; (**c**) 30 min and (**d**) 300 min.

Well-defined and closely packed Au-Ag NWs (Figure 1c and Figure 2a,b) could be synthesized through the reduction of aqueous HAuCl_4_ (0.23 mM) and AgNO_3_ (0.23 mM, 1 mL·h^−1^) with AA (0.5 mM) in the presence of CTAB (9.0 mM) and PPCs (0.005 g) at 30 °C. The time when AA was added into the initial solution was regarded as the initial time. Then the Au-Ag structures in solution at different reductions times 0 min, 10 min, 30 min and 300 min were observed by TEM, and shown in Figure 7. After the addition of AA (Figure 7a), many extremely small nanoparticles were observed in the solution. 10 min later (Figure 7b), some larger nanoparticles were produced on the surface of microorganisms. At 30 min (Figure 7c), some chain-like nanostructures could be obviously observed in the solution, which was observed in the case of Au NWs formation in the previous work [14]. Through the reduction of the precursors for 300 min (Figure 7d), branched nanowires with smooth surface could be observed.

**Figure 8:**
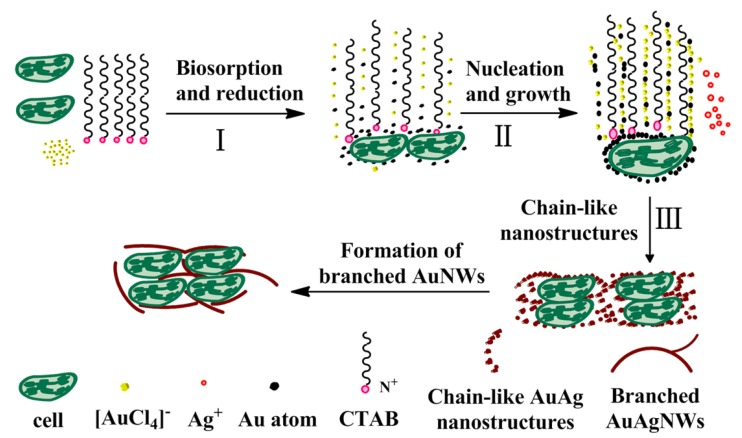
The formation mechanism of Au-Ag nanowires.

As shown in Figure 8, a possible formation mechanism of the Au-Ag NWs was developed. The active sites on the microorganism surface may interact with Au (III) ions. Formation mechanism of Au-Ag NWs could be divided into three stages. In stage I, a small amount of Au (III) ions were first anchored and reduced to Au (0) by some functional groups of the PPC surface [13]. Extremely small Au nanoparticles were produced on the PPCs surface, which were seeds for the follow up reaction. In stage II, after adding AA, the other part of Au (III) and the Ag (I) which was gradually added were reduced to Au (0) and Ag (0), which were the raw material for the growth of Au seeds. With the continuous generation of small Au seeds, Au-Ag nanoparticles were easy to generate because of the extremely similar lattice parameters between Au and Ag. The high-energy planes of these Au-Ag nanoparticles were coated by CTAB [15], and these Au-Ag nanoparticles were rather unstable with a very strong tendency to agglomerate. In stage III, CTAB was not only adsorbed on the high-energy planes of Au-Ag nanoparticles, but also a linear soft template, by which the unstable Au-Ag nanoparticles began to agglomerate linearly. Then long chain-like Au nanostructures were produced due to the direction of CTAB. Gaps among the aggregate Au-Ag nanoparticles were occupied by smaller nanoparticles and the subsequently reduced Au-Ag (0). With the protection of CTAB, the nanoparticles could only grow toward both ends to form nanowires which radial growth was limited [16]. It should be noted that Au seeds at the adjacent dots among PPCs were growth intersection, thus branched Au-Ag NWs were produced. Finally, with a further extension of time, it generated smooth Au-Ag nanowires. Au-Ag NWs tightly wound around PPCs surface, so that they can quickly settle at the bottom of the reaction vessel, thereby to facilitate the separation of Au-Ag NWs from the reaction solution.

### 3.4. Optical Property of Au-Ag NWs

**Figure 9:**
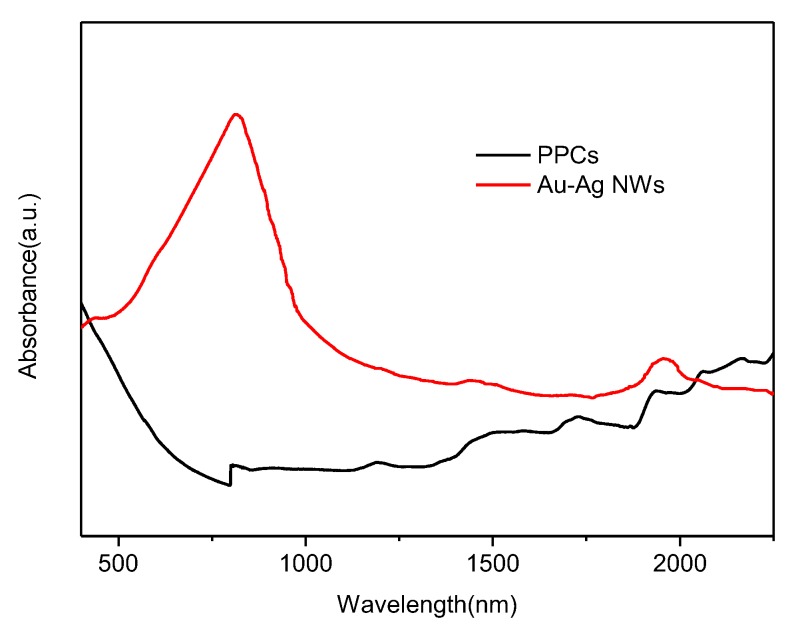
Diffuse reflectance ultraviolet-visible (DRUV-Vis) absorbance spectra of Au-Ag NWs and bare *Pichia pastoris* cells (PPCs).

UV-Vis-NIR spectroscopy and surface-enhanced Raman scattering (SERS) was adopted to examine the optical property of the Au-Ag NWs. As the Au-Ag NWs were closely packed over the PCCs and could not be dispersed in aqueous solutions, their absorption in the liquid phase was not observable. Therefore, solid samples were preferred for Diffuse reflectance ultraviolet-visible spectroscopy (DRUV-Vis) analyses. DRUV-Vis absorbance spectra of the Au-Ag NWs and the PPCs are shown in the Figure 9. The absorbance about 800 nm could be attributed to TSPR of Au-Ag NWs. Moreover, it was evident that there was an intense peak at round 1950 nm in the near-infrared region attributed to LSPR of the Au-Ag NWs, making the Au-Ag NWs potential candidates for functional NIR absorbers. Herein, the Au-Ag NWs were longer than the nanorods and closely packed because of spontaneous aggregation. The LSPR of the Au-Ag NWs was related with the length of nanowires. The absorption peak caused by LSPR shifted to the red with the increasing length of the nanowires. Due to the synergy of Au-Ag, the absorption peak of Au-Ag NWs shifted to the red compared to that [17,18] of Au NWs. In addition, the absorbance at about 800 nm and the intense peak at round 1950 nm in the near-infrared region attributed to LSPR were not observed for the bare PPCs.

**Figure 10:**
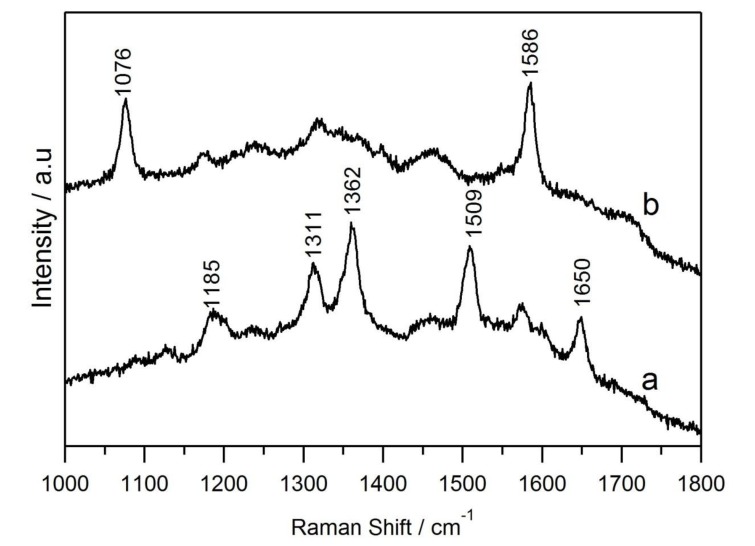
Surface-enhanced Raman scattering (SERS) spectra of (**a**) rhodamine 6G (R6G); (**b**) 4-mercaptobenzoic acid (MBA) on Au-Ag NWs obtained at the laser wavelength of 632.8 nm.

The as-synthesized Au-Ag NWs were employed as surface-enhanced Raman scattering (SERS) substrates, using MBA (10^−6^ M in water) and R6G (10^−9^ M in water) as the target molecules. As shown in Figure 10, five noticeable bands at 1183, 1312, 1363, 1508 and 1650 cm^−1^ were observed for R6G, which can be attributed to the aromatic ring vibrations and the peak positions [19,20]. Two dominant bands at 1076 and 1587 cm^−1^ were observed for MBA, which comes from the aromatic ring vibrations and the peak positions [21]. Corresponding to the Raman bands for different concentrations of the probe molecules, both 10^−6^ M MBA and 10^−9^ M R6G exhibited good SERS signals under the same testing conditions. In contrast, the Raman spectrum at 10^−7^ M MBA and 10^−10^ M R6G did not show any SERS signals (Figure S2). Therefore, the Au-Ag NWs substrate showed strong SERS signals for the MBA and R6G molecules. The characteristic closely packed structure of the Au-Ag NWs endowed them with highly ordered and continuous lattice fringes, but some nano-gaps between the nanowires might be responsible for the significant enhancement of the Raman signals [22].

## 4. Conclusions

In summary, we developed a Microorganism-mediated surfactant-directed (MSD) approach to synthesize closely packed and long Au-Ag NWs in the presence of PPCs and CTAB. Characterization results confirmed that the branched Au-Ag alloy nanowires were polycrystalline. The optimal reaction temperature and AgNO_3_ solution addition rate is 30 °C and 1.0 mL/h, respectively. Interestingly, the Au-Ag NWs herein exhibited a strong absorbance at around 1950 nm in the near-infrared (NIR) region, making the Au-Ag NWs potential candidates as NIR absorbers. In addition, the Au-Ag NWs showed excellent SERS capability for the MBA and R6G molecules.

## Supplementary Materials

The following are available online at http://www.mdpi.com/2079-4991/8/6/376/s1. Figure S1: (A) SEM image of the Au-Ag NWs synthesized using *Escherichia coli* cells (*E. coli*) (B) absence of PPC, Figure S2: SERS spectra of (a) R6G (10^−6^, 10^−10^, 10^−12^ mol L^−1^) and (b) MBA (10^−5^, 10^−6^, 10^−7^ mol L^−1^) in water on Au-Ag NWs obtained at the laser wavelength of 632.8 nm.
