# Supplementary material for: One-Step Synthesis of Au-Ag Nanowires through Microorganism-Mediated, CTAB-Directed Approach
Source: Nanomaterials (Basel). 2018 May 28;8(6):376. doi: 10.3390/nano8060376 (PMC6027277; doi:10.3390/nano8060376)
Supplement: Supplementary file 1 [file nanomaterials-08-00376-s001.pdf]

Article

One-Step Synthesis of Au-Ag Nanowires through Microorganism-Mediated, CTAB-Directed Approach

Luhang Xu [1], Dengpo Huang [1], Huimei Chen [1], Xiaolian Jing [1,*], Jiale Huang [1], Tareque Odoom-Wubah[1,2,*]and Qingbiao Li [2,3]

Cultivation of PPCs and Preparation of Their Powder

The typical conditions are as follows. The medium used for growing PPCs contained yeast extract (10 g L^{-1}), peptone (20 g L^{-1}), and glucose (20 g L^{-1}) in deionized water. After the cells were incubated for 48 h at 30 °C and 150 rpm, they were harvested through centrifugation, and then dried in an oven (60 °C) for 24 h. The dried cells were then crushed into fine powders, which were screened through a 100-mesh sieve. The screened cell powder was stored in a desiccator.

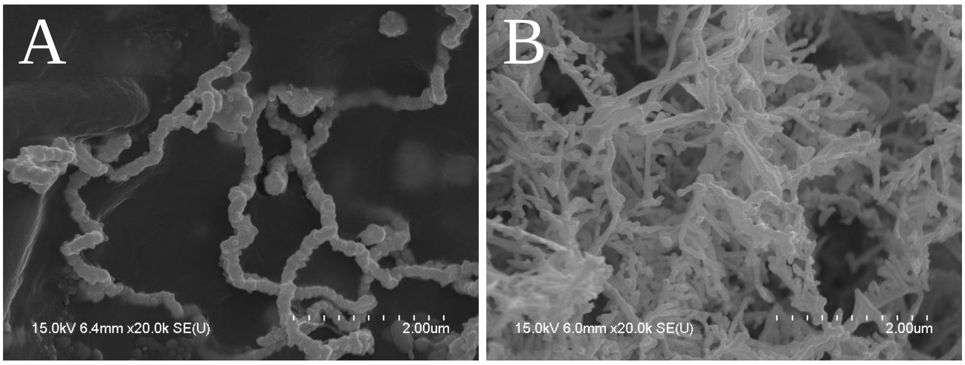

FigS1. (**A**) SEM image of the Au-Ag NWs synthesized using *Escherichia coli* cells (*E.coli*) (**B**) absence of PPC.

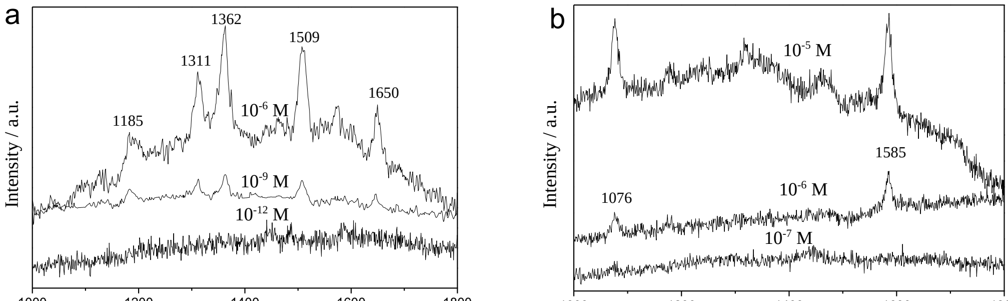

FigS2. SERS spectra of (**a**) R6G (10^{-6}, 10^{-10}, 10^{-12}mol L^{-1}) and (**b**) MBA, (10^{-5}, 10^{-6}, 10^{-7}mol L^{-1}) in water on Au-Ag NWs obtained at the laser wavelength of 632.8 nm.
